# The role of extravillous trophoblasts and uterine NK cells in vascular remodeling during pregnancy

**DOI:** 10.3389/fimmu.2022.951482

**Published:** 2022-07-22

**Authors:** Xiao-Wei Wei, Yu-Chen Zhang, Fan Wu, Fu-Ju Tian, Yi Lin

**Affiliations:** ^1^The International Peace Maternity and Child Health Hospital, School of Medicine, Shanghai Jiao Tong University, Shanghai, China; ^2^Shanghai Key Laboratory of Embryo Original Diseases, School of Medicine, Shanghai Jiao Tong University, Shanghai, China; ^3^Shanghai Municipal Key Clinical Specialty, School of Medicine, Shanghai Jiao Tong University, Shanghai, China; ^4^Institute of Birth Defects and Rare Diseases, School of Medicine, Shanghai Jiao Tong University, Shanghai, China; ^5^Department of Obstetrics and Gynecology, Shanghai First Maternity and Infant Hospital, Tongji University of Medicine, Shanghai, China; ^6^Shanghai Sixth People’s Hospital, School of Medicine, Shanghai Jiao Tong University, Shanghai, China

**Keywords:** spiral artery remodeling, extravillous trophoblast (EVT), uterine natural killer cells, recurrent pregnancy loss (RPL), preeclampsia (PE)

## Abstract

Successful embryo implantation requires both a receptive endometrium and competent blastocysts. After implantation, the maternal decidua undergoes a series of changes, including uterine spiral artery (SA) remodeling to accommodate the fetus and provide nutrients and oxygen for the fetus to survive. Uterine spiral arteries transform from small-diameter, high-resistance arteries to large-diameter and low-resistance arteries during pregnancy. This transformation includes many changes, such as increased permeability and dilation of vessels, phenotypic switching and migration of vascular smooth muscle cells (VSMCs), transient loss of endothelial cells (ECs), endovascular invasion of extravillous trophoblasts (EVTs), and presence of intramural EVT, which are regulated by uterine NK (uNK) cells and EVTs. In this review, we mainly focus on the separate and combined roles of uNK cells and EVTs in uterine SA remodeling in establishing and maintaining pregnancy. New insight into related mechanisms will help us better understand the pathogenesis of pregnancy complications such as recurrent pregnancy loss (RPL) and preeclampsia (PE).

## Introduction

Competent blastocyst implantation is a critical step in the initiation of pregnancy, in which the mother and the semi-allogenic fetus surprisingly coexist peacefully. Successful implantation involves several intricate processes, such as hormonal regulation, the invasion of fetal trophoblasts and well-coordinated maternal decidual immune cell subsets (1). After the implantation, these factors subsequently contribute to uterine spiral artery (SA) remodeling, which includes several processes that are orchestrated by the disruption of vascular smooth muscle cells (VSMCs), the transient loss of endothelial cells (ECs), intravasation or extravasation by interstitial or endovascular trophoblast, and amorphous fibroid deposition containing intramural extravillous trophoblast cells (EVTs) (2). SA remodeling transforms uterine spiral arteries from low-flow, high-resistance vessels to high-flow, low-resistance vessels. Increased blood flow in the intervillous space promotes oxygen and waste exchange between the fetus and mother (3). uNK cells and EVTs are indispensable factors in SA remodeling and they play a vital role in the decidua- or trophoblast-associated remodeling (2). During the process of implantation, there is expansion and activation of uNK cells and EVTs at the maternal-fetal interface (4). Disruptions in either of these populations may contribute to preeclampsia (PE) and recurrent pregnancy loss (RPL) (5, 6). This review focuses on the interaction between uNK cells and EVTs, and their respective impacts on uterine SA remodeling. The pathogenesis of PE and RPL related to dysregulated function of uNK cells and EVTs will also be discussed.

## EVT differentiation and invasion

The blastocyst mainly consists of two structures called the inner cell mass and the trophectoderm, which differentiate into fetus and placenta, respectively (7). During the formation of the placenta, the trophectoderm transforms into mononuclear cytotrophoblasts (CTBs), which form placental villi through branching morphogenesis. In floating villi, which is bathed in maternal blood, CTBs fuse into multinuclear syncytiotrophoblasts (STBs) and form the syncytial layer, where a vast range of functions such as production of pregnancy hormones and clearance of fetal waste products are fulfilled (8, 9). CTBs at branched anchoring villus tips have a proliferative phenotype and differentiate into EVTs, which have an invasive, cytokine-secreting phenotype, forming a stratified structure called the cell column. EVTs in the distal region of the cell column invade the decidua up to the inner third of the myometrium (10). EVTs that migrate into the maternal decidua are called interstitial EVTs and further develop into endovascular trophoblasts that migrate through the spiral arteries (11).

EVT invasion is both stimulated and inhibited by contact with a number of different maternal cell types in the decidua. Lack of decidua may lead to excessive EVT invasion in placenta accreta, while inadequate EVT invasion is associated with pregnancy complications such as preeclampsia (PE) and recurrent pregnancy loss (RPL) (12). Invasive EVT plays an essential role in the SA remodeling process. A number of factors including cytokines, chemokines, and environmental oxygen have been reported to stimulate or inhibit the differentiation/invasion of EVT. CCR1 is expressed on human trophoblasts and its ligands, CCL5 and CCL2, are expressed by decidual tissue. The chemokine-CCR1 system could promote EVT migration and induce the initiation step of trophoblastic invasion toward maternal tissue (13). Some chemokines such as CXCL14 and CXCL6 could inhibit trophoblast invasion through the downregulation of MMP-2 and MMP-9 activity (14, 15). Roser et al. developed a database to predict the interactions between receptors and their respective ligands based on single-cell sequencing of the maternal-fetal interface between 6-14 weeks (16). The researchers confirmed the presence of PDL1 (also known as CD274) in EVTs and identified new inhibitory interactions between KLRB1 (Killer cell lectin like receptor B1) and TIGIT (T cell immunoreceptor with Ig and ITIM domains) on uNK cells and CLEC2D (C-type domain family 2 member D) on EVTs, which suggested that the damaging effects of maternal uNK cells on fetal EVTs was circumvented in the microenvironment of the maternal-fetal interface (16).

Uterine NK (uNK) cells have a significant impact on placentation by regulating the invasion of trophoblasts cells into the decidua basalis and spiral artery remodeling, considering the high abundance of uNK cells in the decidua in the first trimester and their association with EVT through mechanisms including cytotoxicity, local cytokine production or induction of trophoblast apoptosis (17). uNK cells can regulate trophoblast invasion and angiogenesis by releasing IL-8 and interferon-inducible protein-10 upon contact with trophoblasts (18). Coculture of uNK cells with CTB or HTR8 cells significantly promoted trophoblast invasion *via* the secretion of IL-8 and HGF (19). Wang et al. confirmed that IL-22 produced by uNK cells and decidual stromal cells (DSCs) significantly promoted trophoblast proliferation and viability (20). The differential secretion and expression of chemokines/cytokines and their respective receptors could also induce selective leukocyte trafficking to mediate trophoblast invasiveness and placental angiogenesis (21).

Actually, not all species have typical invasive trophoblasts like humans. For example, trophoblast invasion is not extensive in mouse pregnancy, which have a haemochorial placenta with vascular remodeling restricted to the decidua. However, in humans, SA remodeling can extend into the inner myometrium. Similar to human placentation, the rat possesses hemochorial placentation with inherently invasive trophoblasts and EVT-guided transformation of SA remodeling (22). Trophoblast cell invasion is shallow in mice compared with the extensive intrauterine infiltration of rat, thus the rat is regarded as an excellent *in vivo* model for exploring the functions of uNK and EVT (23).

## Phenotypes and subsets of uNK cells during pregnancy

uNK cells constitute the majority of the cell populations at the maternal-fetal interface during the first trimester of human pregnancy. uNK cells account for 70% of local leukocytes, while peripheral blood NK (pNK) cells constitute 15% of circulating leukocytes (24, 25). Given the large number of uNK cells in the decidua during pregnancy, the important role of these cells needs to be further explored. There are also differences in the phenotypes of pNK cells and uNK cells. pNK cells are mainly CD56^dim^ and express high levels of CD16, while uNK cells are mostly CD56^bright^, CD16^-^ cells (26). CD16 is the Fc receptor and can recognize antibody-coated cells and induce NK cells to release cytotoxic cytokines, which is called NK-mediated antibody-dependent cellular cytotoxicity (27). CD56^bright^, CD16^-^ NK cells secrete cytokines such as TGF-β while CD56^dim^, CD16^+^ NK cells are more cytotoxic (28). The main function of uNK cells is to release cytokines/chemokines that induce angiogenesis and placentation, while the main function of pNK cells is immune defense against infections and tumors through their cytolytic activity and production of cytokines, such as IFN-γ and TNF (29). Granule content and organization of uNK cells are different from those of pNK. Although uNK cells are poorly cytotoxic, they have large granules filled with perforin and granzymes. Meanwhile, pNK cells from healthy individuals are more cytotoxic and produce more cytokines with the increase of granule size (30). Granules in uNK cells are significantly larger in size and fewer in number compared with those in pNK cells (31, 32).

Diverse subsets of NK cells are dependent on different marker genes expressed on cells or diverse cytokine profiles in both humans and mice. A study using flow cytometry showed the production of type 1, type 2, type 3 and regulatory cytokines in uNK cells (33). uNK cells were grouped as NK1, NK2, NK3 and NKr1 according to the different cytokines they released. NK1 cells are Th1 (Type 1 T helper cells) cytokine-producing cells while NK2 cells release Th2 (Type 2 T helper cells) cytokines. TGF-β is produced by NK3 cells and IL-10 is released by NKr1 cells (34). Similar to the Th1/Th2/Th3/Tr1 (Type 1 T regulatory cells) paradigm, which plays an important role in balancing T cell-mediated immune stimulation and immune tolerance, the NK1/NK2/NK3/NKr1 paradigm is critical in the maintenance of normal pregnancy (35). Immature NK2 cells, which produce IL-13 and IL-5, eventually develop into NK1 cells that release IFN-γ. NK3 cells are the central population of uNK cells. The number of decidual NK3 cells and NKr1 cells that can produce TGF-α-related immunosuppressive factors is increased during normal pregnancy but decreased in miscarriage (33). In contrast, the number of decidual NK1 cells is increased in patients with miscarriage. The subpopulation of NK cells is changed from an NK3-predominant state to an NK1-predominant state in patients with miscarriage (33).

Yang et al. divided uNK cells into cytotoxic NK cells, tolerant NK cells, regulatory NK cells and memory NK cells (36). Cytotoxic NK cells are mainly CD27^-^CD11b^+^ NK cells, which play an essential role against infection at the maternal-fetal interface during pregnancy. Memory NK cells are termed pregnancy-trained decidual NK (PTdNK) cells and have high expression of LILRB1 and NKG2C. The incidence of placental dysfunction decreased during repeated pregnancies compared with that in first pregnancies, which may be partly attributed to the activation of PTuNK cells. VEGFα released by PTuNK cells can promote vascular remodeling (37). Regulatory NK cells can secrete cytokines and chemokines, such as CD49a^+^Eomes^+^ NK cells. Tolerant NK cells are immature cells that may eventually develop into other subsets of NK cells (36).

## KIR/HLA interactions

uNK cells have been reported to be involved in the guidance of trophoblast invasion as well as uterine SA remodeling during pregnancy *via* direct interaction between ligands and receptors or cytokine production (38). KIRs are paired receptors expressed on NK cells with both activating and inhibitory functions. Most KIRs are inhibitory, so that they were initially thought as inhibitory receptors and named “Killer-cell Inhibitory Receptors”. When A limited number of activating receptors within this family were found, both activating and inhibitory groups were termed “killer-cell immunoglobulin-like receptors” (KIRs) (39). The groundbreaking “missing self” hypothesis, firstly put forward by Karre and Ljunggren, stated that inhibitory KIRs could recognize self-major histocompatibility (MHC) class I surface molecules and protect the target cells against the cytotoxic activity of NK cells, which is essential in facilitating self-tolerance (40, 41). The KIR gene family is currently composed of 15 genes (KIR2DL1, KIR2DL2, KIR2DL3, KIR2DL4, KIR2DL5A, KIR2DL5B, KIR3DL1, KIR3DL2, KIR3DL3, KIR2DS1, KIR2DS2, KIR2DS3, KIR2DS4, KIR2DS5, KIR3DS1) and 2 pseudogenes (KIR2DP1 and KIR3DP1), which are named based on the number of Ig-like domains and the length of the cytoplasmic tail (42). Generally, inhibitory receptors have a long cytoplasmic tail and are labeled as “L”, while activating receptors have a short cytoplasmic tail and are labeled as “S”. Only the KIR2DL4 receptor is exceptional and have both activating and inhibitory signals (43).

Human leukocyte antigen (HLA), also known as major histocompatibility complex (MHC), are the most polymorphic loci in the human genome, encoding the human MHC class Ia (HLA-A, HLA-B, and HLA-C), class Ib (HLA-E, HLA-F, HLA-G and HLA-H) and class II (HLA-DR, DQ, DM, DO and DP) molecules (44). EVT express non-classical HLA-G, HLA-E, HLA-F and polymorphic HLA-C molecules instead of the typical class I or class II molecules in normal pregnancy. Meanwhile, other trophoblast cell types have no expression of HLA genes (45, 46). HLA-C allotypes, including the C1 or the C2 epitope, act as ligands for KIRs. Inhibitory KIR2DL1 receptors and activating KIR2DS1 receptors bind to the C2 epitope, while inhibitory KIR2DL2/3 receptors bind to the C1 epitope (47, 48). The binding strength of inhibitory receptors is higher than that of activating receptors upon binding to the same epitope. HLA-G are unusual ligands binding to KIR2DL4 (49). HLA-F can be expressed in two ways on the cell surface, including open conformers binding to KIR3DS1 and KIR3DL2 on NK cells, as well as peptide-bound HLA-F binding to the leukocyte immunoglobulin-like receptor (LIR) family, ILT2 and ILT4 (50, 51). HLA-F can also be expressed intracellularly in leukocytes (52). Disrupted interactions of KIR expressed on uNK cells with HLA expressed on EVT may disturb the balance between immune defense and immune tolerance and contribute to pregnancy complications (53, 54).

With the development of technology, Vento-Tormo et al. used nearest-neighbor cluster analysis of single-cell transcriptomics from first trimester decidua to redefine uNK cells into three major subsets including uNK1, uNK2 and uNK3, which all co-express the tissue-resident markers CD49a and CD9 (55). uNK1 is the only subset that express LILRB1, which is the receptor binding to HLA-G molecules expressed on EVT. Inhibitory receptors KIR2DL1, KIR2DL2 and KIR2DL3 and activating receptors KIR2DS1 and KIR2DS4 are KIRs that have high affinity for HLA-C and have higher expression levels in the uNK1 subset. The HLA-E receptors, including activating NKG2C and NKG2E as well as inhibitory NKG2A, are specifically expressed on uNK1 and uNK2 cells instead of the uNK3 subset. uNK1 cells secrete higher levels of CSF1, which bind to the receptor CSF1R expressed on EVT and macrophages. The above results suggest that uNK1 play a vital role in interacting with EVT and responding to EVT (55). uNK2 and uNK3 secrete more XCL1 chemokines than uNK1, which have interactive receptors both on maternal dendritic cells and fetal EVT (32). uNK3 cells express high levels of CCL5, while CCR1, the receptor for CCL5, is expressed on EVT. The expression pattern of the CCL5-CCR1 ligand-receptor complex suggests that uNK3 may also play a role in regulating EVT invasion (13).

Innate lymphoid cells (ILCs) are effectors and regulators that play a major role in innate immunity and tissue modeling and repair (56). Uterine NK cells are the best characterized member of the ILC family, which are detectable in decidua in the early phases of pregnancy (57). Huhn et al. have identified five main subsets of ILCs with different functional activities and diverse capacities to produce cytokines during first trimester using mass cytometry, including uterine NK cells (uNK)1-3, ILC3 and proliferating NK cells (32). They also demonstrated that uNK1 had lower responses in missing self assays, but higher responses to stimulation by cross-linking activating KIR2DS4, which may be associated with changes in granule content and organization followed by increased KIR expression in uNK1 (32, 55, 58). Further, Vazquez et al. found two novel decidual ILC subsets (C10 and C2) that express low T-bet and divergent Eomes levels through dimensionality reduction coupled with clustering using term human decidua samples (59). Combined with data of recent single cell sequencing of first trimester decidua (55), they proposed the continuity of decidual ILC subsets across pregnancy from early pregnancy through late gestation (59).

## The role of uNK cells in uterine spiral artery remodeling

Vascular remodeling is a crucial step in the establishment and maintenance of pregnancy. Previous studies have shown that SA remodeling mainly depends on EVT (60, 61). However, some other evidence demonstrates that uNK cells dominate the initiation of the remodeling process, while EVTs play a secondary role (62, 63). Early uterine SA remodeling only includes the process of extracellular matrix degradation, transient loss of vascular ECs, and vascular smooth cell separation (2). This process is called decidua-associated remodeling, followed by trophoblast-associated remodeling (2). “Decidua-associated” or “Trophoblast-independent” remodeling is a maternal-mediated phase that precedes EVT invasion (2). Endothelial swelling and separation of medial VSMCs occurred in partially remodeled SA without intramural or endovascular EVTs, suggesting an early stage of SA remodeling prior to invasion of vessel wall by EVTs (64). Further study in pseudo-pregnant mice showed that early SA remodeling was independent of EVTs. Compared to normal pregnancy, disappearance of EVTs in pseudo-pregnant mice did not disturb early SA remodeling (65). The accumulation of uNK cells at the maternal-fetal interface during pregnancy suggests that uNK cells play an important role in the process of uterine SA remodeling. However, the exact function of uNK cells in SA remodeling is not fully understood. The possible role of uNK cells in SA remodeling may be to mediate the disorganization and clearance of VSMCs and endothelial cells (66).

The proportion of uNK cells in the first trimester is significantly higher than the proportion of uNK cells at term pregnancy (67, 68). uNK cells are an important source of growth factors and cytokines (18, 69). Therefore, uNK cells have two main functions, including promoting vascular remodeling and regulating trophoblast invasion (18, 70). Robson et al. demonstrated that uNK cells from early human pregnancy can induce morphological changes of VSMCs and breakdown of extracellular matrix component (71). The primary role of VSMCs is to control vessel contraction, thus regulating blood pressure and blood vessel tone. During the process of partially SA remodeling, VSMCs transformed from a contractile phenotype to a more synthetic or dedifferentiated phenotype. Markers of contractile phenotype are α-SMA, calponin, H-cal, MyHC and smoothelin, while the synthetic marker is osteopontin (64). VSMCs exhibit plasticity and can dedifferentiate in response to changes in local environmental cues including cell-cell and cell-matrix interactions and various inflammatory mediators during vascular development or vascular injury (72, 73).

uNK cells surround the spiral arteries and secrete many factors such as Angiopoietin (Ang) -1, Ang-2, VEGF-C, and IFN-γ, which can disrupt the integrity of VSMC and mediate extracellular matrix degradation (71). uNK cells can also secrete a variety of metalloproteinase (MMP) such as MMP2 and MMP9, which could degrade gelatin and break down collagen in the spiral artery model (74). A recent study showed that when VSMCs were cultured in uNK cell conditioned media for 24 h, the expression of VSMC markers such as calponin, smooth muscle protein 22-alpha (SM22α), smooth muscle alpha actin (αSMA), and myosin heavy chain 11 (MYH11) was significantly inhibited, suggesting that active uNK cells could affect VSMCs dedifferentiation. The dramatic differentiation of VSMCs eventually contributes to the loss of VSMCs, which is an important step in SA remodeling (75). IFN-γ derived from uNK cells could upregulate the LncRNA Maternal expressed gene 3 (MEG3) and increase the expression of MMP-2, thus promoting VSMCs apoptosis and migration (76). A subsequent study showed that miR-361-5p targeted by lncRNA MEG3 played an important role in inhibiting VSMCs proliferation and promoting VSMCs apoptosis (77).

## The role of EVTs in uterine spiral artery remodeling during pregnancy

It has been proposed that the remodeling of both VSMCs and ECs was mediated by apoptosis through Fas signaling pathway or apoptotic cytokines (78–80), but Bulmer et al. demonstrated that SA remodeling involved the disorganization, rounding and migration of VSMCs in an EVT-dependent manner, instead of the apoptosis of VSMCs (81). Further, others have demonstrated that VSMCs only undergo apoptosis once they have undergone phenotypic switching and migrated away from the vessel wall, both processes require EVTs (64, 71, 82, 83). The uNK cells attract EVT toward the vessels through angiogenic growth factors and cytokines, thus attracting the VSMCs away from the vessel wall into the decidual stromal, where they undergo apoptosis and are phagocytosed by decidual macrophages (82). Upregulated MEG3 in trophoblasts could induce VSMC apoptosis and impair trophoblast migration by inactivating the PI3K/Akt pathway, suggesting that MEG3 could inhibit SA remodeling by EVT-mediated VSMCs loss and inhibiting EVT invasion (84). In addition, exosomes derived from EVTs also have many effects on SA remodeling and immune balance. Exosomes may promote the migration of VSMCs out of vessel walls and lead to uterine SA remodeling (85).

It was previously believed that EVTs completely replaced the endometrium in SA remodeling and acquired endothelial cell (EC) - like characteristics (86, 87). However, a comprehensive review by Pijnenborg et al. questioned the existing dogma of ECs mimicry by EVTs and demonstrated that EVTs and ECs coexisted in a fully remodeled SA with double labeling immunohistochemistry (2). Bulmer et al. further demonstrated that there was no evidence that EVTs replaced the endothelium of the vessel wall and ECs were never completely lost in the vessels. It was the presence or absence of an endovascular EVT plug that partly determined the extent of coverage of the SA lumen by ECs (88). In the presence of endovascular EVTs, ECs undergo morphological change and become rounded and discontinuous, which morphology is similar to EVT. This may explain that why ECs are mislabeled as EVTs. In fully remodeled SA, ECs showed a more typical morphology, which are elongated and flattened against the underlying extracellular matrix (ECM) (88).

The role of EVTs play in the completion of arterial remodeling is also highly dependent on the ability of migration/invasion into the decidual stroma. Inadequate trophoblast migration and invasion or induced trophoblast apoptosis and decreased proliferation disrupt angiogenesis and vascular remodeling. Our team has also done a series of work in terms of trophoblast invasion and migration. We firstly identified the transcription factor YY1 (Yin Yang 1), which could promote trophoblast invasion by targeting matrix metalloproteinase-2 (89). Besides we also revealed that EIF5A1 (Eukaryotic translation initiation factor) could promote trophoblast migration and invasion by binding directly to ARAF mRNA, thus activating the integrin/ERK signaling pathway (90).

uNK cells can regulate SA remodeling indirectly *via* modulating EVT invasion. Pollheimer et al. (91) revealed that the pro-invasive effect of uNK cells on EVTs was in a stage-dependent way. The conditioned media of uNK cells harvested from later gestational age within the first trimester (10-13 weeks’ gestation) could promote EVT invasion, while uNK cell-derived conditioned media from an earlier developmental stage did not have an effect on EVT invasion. This can be reflected by another experiment showing that the number of circulating EVTs peaked in 12-13 gestational weeks (92). EVTs with strong invasive ability invade the uterine arteries and form plugs in the lumen of the uterine arteries, which eventually disintegrated and migrate into the maternal blood. The peaked number of EVTs in 12-13 gestational weeks may be related to the pro-invasive effect of uNK cells on EVTs in 10-13 gestational weeks.

## Cell-to-cell crosstalk between uNK cells and EVTs during pregnancy

The direct or indirect interaction of uNK cells with EVT has been shown to facilitate the induction and maintenance of immune tolerance, protect the placenta against pathogen infection, and promote SA remodeling (58, 93, 94).

### Immune tolerance

Fetuses carrying paternal human leukocyte antigen (HLA) are semi-allogenic to mothers. However, they coexist peacefully. Fascinating adaptations both on maternal or fetal side rely on fine-tunings of decidual immune cells, decidual stromal cells, and trophoblasts to direct away from immune rejection towards immune tolerance (95). Maternal-fetal interface is important during the establishment of pregnancy-associated immune homeostasis. MHC molecules such as HLA-C, -G, and -E expressed by EVTs have been demonstrated to be ligands of either activating or inhibitory uNK cell receptors, providing a delicate balance between tolerance of trophoblast from maternal immune response and cytolytic activation of uNK (96). HLA-G is mainly expressed in EVT and believed to be a critical factor to prevent maternal immune attack to semi-allogenic fetus. Upon encountering fibronectin, which is a major component of uterus, EVT increased the expression of HLA-G. HLA-G^+^ EVT did not elicit a profound cytokine response by uNK, thus HLA-G^+^ EVT has the greater ability to induce maternal tolerance compared with HLA-G^-^ EVT (97). HLA-G is also expressed either on the surface or inside of the uNK. uNK containing surface HLA-G account for about 2.5% of the total number of uNK and they acquire surface HLA-G through forming synapses with HLA-G^+^ EVT. When virus attacked, uNK were activated and resulted in the disappearance of internalized HLA-G as well as restoration of cytotoxicity. The interaction of EVT with uNK formed a HLA-G cycle of trogocytosis, endocytosis and degradation, which process contributed greatly to immune tolerance and antiviral immunity (98). Although HLA-G has been proved to be related to the induction of immune tolerance, polymorphic HLA-C can elicit a maternal immune response. It has been reported that engagement of activating KIR with HLA-C can reduce the risk of pregnancy complications, possibly through providing specific immunity to viral and bacterial pathogens (48). Specifically, interaction of KIR2DS1 with HLA-C2 contributes to uNK activation, while interaction of KIR2DL1 with HLA-C2 leads to inhibition of cytotoxicity of uNK cells (99). NKG2A could engage with its ligand HLA-E to regulate placental function and immune adaptation, thus inhibiting preeclampsia (PE) occurrence in human and mice (100).

The expression of HLA-C, G, E and F is distinct in three trimesters of pregnancy, suggesting that different types of EVT have distinct ability to interact with uNK cells and influence NK cell maturation and function (101–103). The expression of HLA-C is highest in the first trimester, while the expression of HLA-G is highest in the term pregnancy. HLA-E is only expressed in the first trimester (46). Only the first trimester EVT, instead of the term trimester EVT, upregulated the cell surface expression levels of HLA-C and HLA-G in response to the stimulation of the proinflammatory cytokine IFN-γ (103). Besides, different levels of HLA-E and HLA-G may regulate uNK responses by influencing the expression of HLA-E receptors (NKG2A/C) and HLA-G receptors (KIR2DL4, LILRB1 and LILRB2) (103). HLA- F is highly expressed on normal EVT of the first trimester placenta and becomes intracellular and weaker in the second and term trimester (46). This result is totally contrary to an earlier study, which showed that there was an increase in expression of HLA-F from the second trimester to term trimester and HLA-F was expressed only in the cytoplasm during the first trimester, after which HLA-F moved to the cell surface with the progression of pregnancy (104). This controversy needs to be further investigated. Zhang et al. found that there was an increased number of EVTs around uNK cells in the first trimester compared to the second trimester and EVTs could decrease the activating receptor NKG2D on uNK cells (105). In the second trimester, uNK cell function was inhibited through the loss of interactions between uNK cells and EVTs, which contributed to immune tolerance (105).

### Immune defense

Placenta is not only an organ that provides nutrients and exchanges oxygen and gas between fetus and mothers, it is also a powerful physical barrier to prevent transmission of virus and microorganism by initiating innate immune response. A strong association between intrauterine pathogen infection and pregnancy complications such as preterm birth, preeclampsia and abortion has been demonstrated by several studies (106–108). Epidemiological evidence shows that pregnant women are more susceptible to viral infection possibly due to immune tolerance (109).

Upon encountering cytomegalovirus (CMV)-infected decidual stromal cells, uNK cells change phenotypes and become more cytotoxic through both the NKG2D and the CD94/NKG2C or 2E activating receptors, which increases antiviral immune responses (110). A study has also demonstrated that uNK expressing activating KIR2DS1 acquired higher cytotoxic function when exposed to human cytomegalovirus (HCMV)-infected decidual stromal cells (DSC), especially when DSCs express HLA-C2 (58). However, CMV also resides and replicates in the trophoblasts (111, 112). In contrast to infected DSC, uNK were unable to secrete cytotoxic cytokines or degranulate upon encountering with CMV-infected primary EVT (58). Interestingly, a significant loss of HCMV-infected EVT was observed upon coculture with uNK even without cytokine production or degranulation (58). This phenomenon was further explained by a recent study, which demonstrated that uNK cells killed intracellular bacteria through transferring Granulysin to EVT *via* nanotubes without killing EVT, which is independent of degranulation and cytokine secretion (94). Although uNK-secreted growth factors and cytokines are important factors to defense infection and facilitate placentation, direct transfer of cytosolic proteins and nutrients through nanotubes could also contribute to uNK regulation of placentation.

### SA remodeling

SA remodeling is a crucial process during pregnancy to provide enough blood supply to meet the demands of the growing fetus. The surrounding niche cells including uNK and EVT and uterine environment including hormones and oxygen tension play an important role in SA remodeling during pregnancy. *In vitro* experiments of VSMC cell line demonstrated the high efficiency of uNK and EVT in inducing VSMCs dedifferentiation (113).

The endocrine system and the immune system act synergistically during implantation and maintenance of pregnancy. For example, studies in mice show that uNK cells play an important role in the modification of uterine blood vessels *via* an IFN-γ pathway. The IFN-γ production of uNK cells could be inhibited *via* glucocorticoid receptor, which is cross-reacted with progesterone, further inducing immune tolerance during pregnancy (114, 115). Human decidual stromal cells will upregulate the expression of IL-15 mRNA during progesterone-induced decidualization, which may influence uNK proliferation, differentiation and production of cytokines (116). Estrogen also plays an important role in regulating the functions of human uNK cells through mediating uNK cell migration and promoting secretion of CCL2 from uNK cells, which facilitates uNK cell-mediated angiogenesis (117). The HLA-F mRNA expression level is upregulated under the stimulation of progesterone, while knockdown of the progesterone receptor downregulates HLA-F expression level, suggesting a role of progesterone in regulating HLA-F expression and EVT invasion, further impacting the process of SA remodeling (118).

In both humans and rodents, the primary role of uNK cells is in regulating early stages of spiral artery remodeling, which increases blood supply to the placenta and is essential to set the foundation for maternal nutrient and oxygen delivery to the placenta for fetal growth. Blood flow to the human intervillous space does not begin until 10 to 12 weeks of pregnancy (119). Placental PO2 values measured in the 12-13 weeks were significantly increased compared with those obtained at 8-10 weeks, which suggest that the increase of placental PO2 at the end of the first trimester is related to the establishment of continuous maternal blood flow in the intervillous space (120). The change of oxygen tension at the maternal-fetal interface is thought to have secondary influence on EVT invasion. Physiologically hypoxic conditions in the first trimester, which is 2%-3% oxygen, are believed to promote embryo implantation and trophoblast invasion compared to normoxic conditions (121). The above results show that uNK cells have the potential to indirectly regulate trophoblast invasion and SA remodeling through regulation of oxygen tension at the maternal-fetal interface.

**Figure 1:**
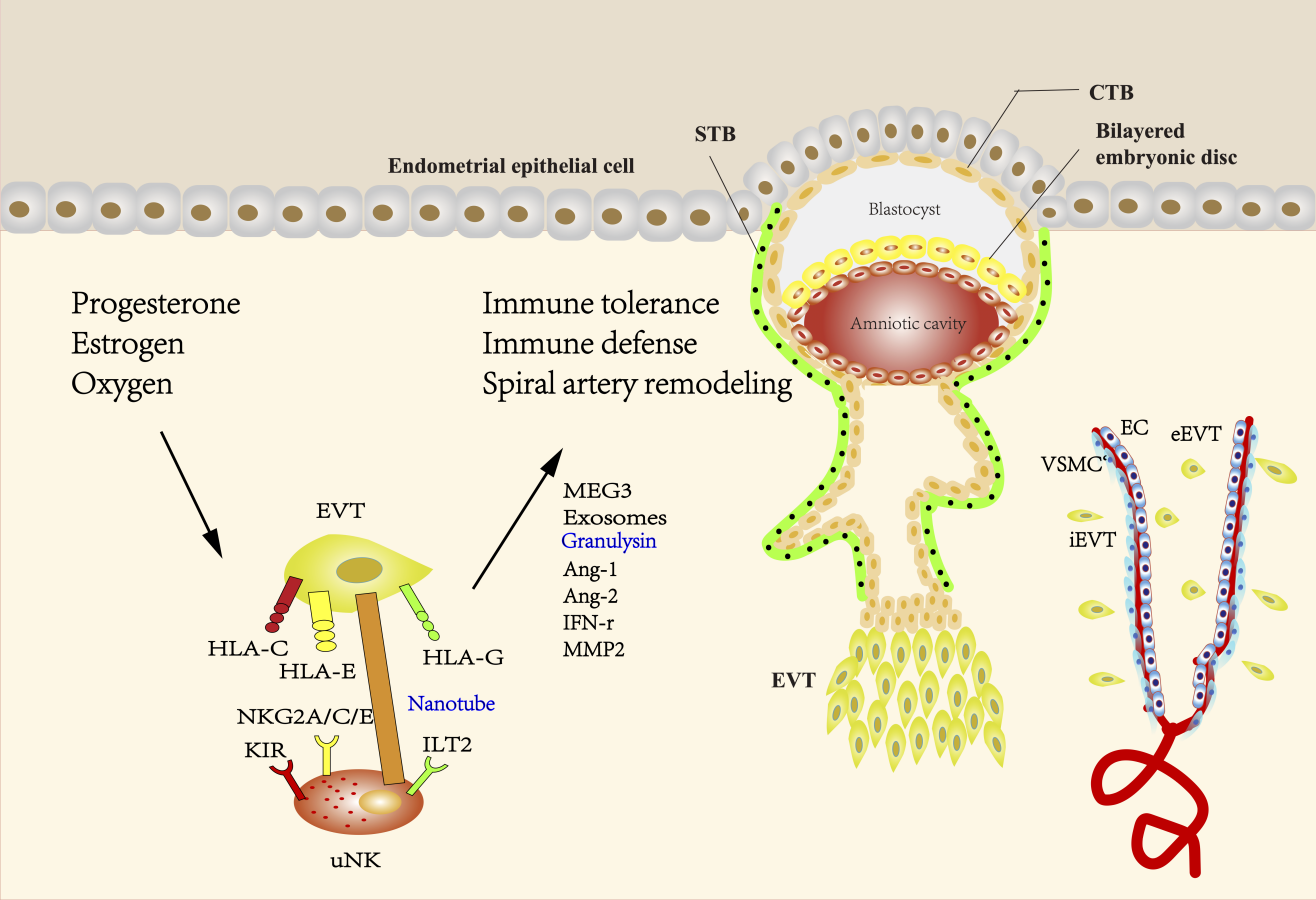
The interaction between EVT and uNK cells during pregnancy. The blastocyst mainly consists of two structures called the inner cell mass and the trophectoderm, which differentiate into fetus and placenta, respectively. During the formation of the placenta, the trophectoderm transforms into mononuclear CTBs, which have two differentiation ways. CTBs can fuse into multinuclear STBs and form the syncytial layer. Proliferative but non-invasive CTBs can switch to invasive but non-proliferative EVT, forming a stratified structure called the cell column. EVTs that migrate into the maternal decidua are called interstitial EVTs (iEVT) and further develop into endovascular trophoblasts (eEVT) that migrate through the spiral arteries. The direct or indirect interaction of uNK cells with EVT has been shown to facilitate the induction and maintenance of immune tolerance, protect the placenta against pathogen infection, and promote SA remodeling. Uterine environment including hormones and oxygen tension at the maternal-fetal interface plays an important role in regulating EVT and uNK cells during pregnancy. HLA-C, -G, and -E expressed by EVTs have been demonstrated to be ligands of either activating or inhibitory uNK cell receptors, providing a delicate balance between tolerance of trophoblast from maternal immune response and cytolytic activation of uNK. HLA-G is mainly expressed in EVT and believed to be a critical factor to prevent maternal immune attack to semi-allogenic fetus, while polymorphic HLA-C can elicit a maternal immune response. The interaction between HLA-G and ILT2 can also contribute to the release of proangiogenic factors. Engagement of activating KIR with HLA-C can reduce the risk of pregnancy complications, possibly through providing specific immunity to viral and bacterial pathogens. NKG2A/C/E could engage with its ligand HLA-E to regulate placental function and immune adaptation. uNK cells surround the spiral arteries and secrete many factors such as Ang-1, Ang-2, and IFN-γ, which can disrupt the integrity of VSMC and mediate extracellular matrix degradation. IFN-γ derived from uNK cells could upregulate the MEG3 and increase the expression of MMP-2, thus promoting VSMCs apoptosis and migration. Exosomes derived from EVTs may promote the migration of VSMCs out of vessel walls and lead to uterine SA remodeling. uNK cells can transmit granulysin into EVT *via* nanotubes and kill intracellular bacterial without damaging EVT. (uNK cells, uterine natural killer cells; EVT, extravillous trophoblast; CTBs, cytotrophoblasts; STBs, syncytiotrophoblasts; iEVT, interstitial EVTs; eEVT, endovascular trophoblasts; SA, spiral artery; HLA, human leukocyte antigen; ILT2, Ig-like transcript 2; KIR, Killer-cell immunoglobulin-like receptors; NKG2A/C/E, CD94/NK group 2 member A/C/E; Ang-1, Angiopoietin-1; IFN-γ, interferon-γ; VSMC, vascular smooth muscle cell; MEG3, maternally expressed 3; MMP2, matrix metalloproteinase-2).

A study suggested that placental vascularity might depend on the allogeneic interaction between maternal KIR on uNK cells and paternal HLA-C expressed by trophoblasts. The interaction induces the secretion of proangiogenic factors including Ang-1 and Ang-2 and the release of proinflammatory cytokines, such as IL-8, IL-10, interferon-γ (IFN-γ), tumor-necrosis factor-α (TNF-α) and macrophage inflammatory protein (MIP) (18). The interaction between HLA-G and ILT2 could also contribute to the release of proangiogenic factors (122). HLA-G expression could be inhibited by miR-133a, which might impair the angiogenic and invasive functions of uNK cells (123). It has also been demonstrated that uNK cells can transform into a senescent phenotype upon interacting with HLA-G from trophoblasts during pregnancy (122, 124). The NK cells of senescent phenotype would produce pro-angiogenic factors that regulate trophoblast invasion and spiral artery remodeling (125). HLA-F is highly expressed on the surface of invasive EVT both ex vivo and *in vitro*. The expression of HLA-F on the surface of invasive EVT is increased compared to that of the non-invasive proliferating EVT, suggesting a role of HLA-F in EVT invasion and SA remodeling (46, 126) (**Figure 1**).

## Dysregulated uNK cell and EVT functions in the pathogenesis of RPL

RPL is a critical reproductive complication. An individual with this disease suffers at least two consecutive pregnancy losses. RPL has relatively high morbidity and affects approximately 1% to 2% of women (127). Common causes of pregnancy loss include anatomical anomalies and genetic, endocrine and immunological factors. However, the reasons for almost half of the cases remain unexplained (128). A lack of inhibition of uNK cells and dysregulated EVT migration and invasion may partly contribute to the occurrence of RPL (129).

Omnia et al. found an abnormal increase in both CD158b (inhibitory KIR) and CD161 (activating KIR) among all NK cell subsets in the peripheral blood and decidua of patients with recurrent pregnancy loss. The cytotoxic function of CD16^+^ NK cells is related to the expression of CD161 (130). These findings confirmed the hypothesis of Zhu et al. that the immune system is disturbed by increased expression of inhibitory and stimulatory KIRs in patients with miscarriages (131). The expression of HLA-G is decreased in women with RPL (132). A recent finding by Zhou et al. showed that EVT-derived HLA-G interacted with ILT2 in uNK cells and promoted the expression of PBX1, which is a transcription factor with critical functions. PBX1 is mainly expressed in CD49a^+^ tissue resident NK cells and can promote the transcription of growth-promoting factors (GPFs) that contribute to fetal development. In addition, there was a decrease in the number of NK cells in PBX1-mutant mice, suggesting that PBX1 might indirectly modulate the immune microenvironment by downregulating uNK cells. Impaired CD49a^+^PBX1^+^ uNK cells may be related to an increased risk of RPL (133). Consistently, Li et al. (134) found lower levels of CD49a^+^ in patients with RPL than in healthy controls. However, numerous studies have found that there was an increased population of uNK cells in the luteal-phase endometrium of women with RPL (135–138). An increase in uNK cells and their production of cytokines and angiogenic factors promoted endometrial angiogenesis, which could lead to increased level of oxygen, thus increasing oxidative stress (139, 140). Consistently, Chen et al. found that the number of micro-blood vessels in the decidual tissues of patients with RPL during the peri-implantation period were increased compared with those of the normal fertility group (141). Based on the above evidences, we propose that different numbers of uNK cells in the endometrium before and after pregnancy have a distinct effect on the pathogenesis of RPL.

Although the interaction between KIR and HLA-C is critical in understanding the role of NK cells in RPL, nonspecific MHC class I inhibitory receptors such as TIM-3 (T-cell immunoglobulin and mucin domain-3) also partly explain the occurrence of RPL (142). TIM-3 is expressed on the surface of pNK cells and could induce the secretion of anti-inflammatory cytokines and promote immune tolerance at the maternal-fetal interface. Patients with RPL have a decreased percentage of TIM-3^+^ CD56^+^ cells in the peripheral blood (143). Besides, studies in human and mice showed that autophagy levels might affect the process of placentation through regulating trophoblast invasion and NK cell residence in the decidua, which provided new insight into the treatment of RPL (144, 145). Tan et al. revealed that suppressed trophoblast autophagy increased the secretion of IGF-2 (Insulin growth factor-2), which induced the differentiation of NK cells from a less cytolytic phenotype to cells with high killing activities. IGF-2 could also downregulate PEG10 (paternally expressed 10) levels, which inhibited trophoblast invasion. Moreover, autophagy suppression disturbs the function of memory uNK cells during the first pregnancy, which could attack newly formed trophoblasts during subsequent pregnancies and increase the incidence of RPL (146).

## Dysregulated uNK cell and EVT functions in the pathogenesis of PE

PE is a common pregnancy complication due to poor placental development (147). PE clinically manifests as proteinuria and new-onset hypertension after 20 weeks of pregnancy. There are two types of PE, including early-onset (before 34 weeks) and late-onset (after 34 weeks). PE is the main reason for the morbidity and mortality of mothers and fetuses (148). However, the pathogenesis of PE remains unclear.

PE progression is divided into two stages: placental ischemia derived from disrupted SA remodeling in the first trimester and subsequent inflammation and hypertension resulting from excessive release of antiangiogenic factors such as sENG (soluble endoglin) and sFLT1 (soluble fms-like tyrosine kinase 1) in the second and third trimesters (149, 150). It is speculated that the changes in the number and subsets of uNK cells and inadequate trophoblast invasion may damage SA remodeling and further contribute to the occurrence of PE (151).

Zhang et al. found that the number of CD56^+^ CD3^-^ uNK cells was increased, and activation markers on uNK cells such as IFN-γ (interferon gamma), IL-8 and CD107a were obviously dysregulated in PE (152). The expression of TGF-β in the decidua of patients with PE was negatively correlated with VEGF levels and activation markers expressed on uNK cells (152). Thus TGF-β might play a critical role in the pathogenesis of PE. Another study showed that the reduced population of uNK cells was associated with the occurrence of PE through an altered cytokine environment, which might lead to defective trophoblast invasion and SA remodeling (153). However, it was found that insufficient perfusion of uterine arteries could stimulate the activation of cytolytic NK cells, while reduced number of NK cells could rescue placental ischemia-induced damage such as hypertension and inflammation (154).

The state of SA remodeling can be reflected by uterine artery Doppler ultrasound. When SA remodeling is damaged, patients will have decreased maternal blood supply and high blood flow resistance. Thus impaired SA remodeling is represented by a high resistance index while normal SA remodeling is represented by a normal resistance index. Fraser et al. (80) found that the pro-invasive effect on EVTs by uNK cells from women with high resistance indexes was decreased compared with those from women with average resistance indexes. This is consistent with the findings of Wallace et al (5). Besides, uNK cells from women with high resistance indexes may secrete fewer pro-apoptotic factors and were less likely to induce VSMC apoptosis compared with those from women with normal resistance indexes (80).

## Conclusion

The immunology of the maternal-fetal interface is extremely complex, because there are great variety of participating components and diverse interactions between fetal and maternal cells. There are still huge gaps in understanding the role of uNK cells and EVTs in SA remodeling and the interaction between uNK cells and EVTs, dysfunction of which may result in the pregnancy-related diseases. This review highlights the critical and important role that uNK cells and EVT play in spiral artery remodeling during pregnancy. Several problems remain to be resolved in the future.

1. uNK cells degranulate and secrete cytokines in response to CMV-infected decidual stromal cells, while uNK cells do not have such effect on CMV-infected EVT. Further study showed that uNK cells transmitted granulysin into EVT *via* nanotubes and kill intracellular bacterial without damaging EVT. The balance between immune tolerance and immune defense against bacterial or virus in the placenta during pregnancy needs to be further explored.

2. With the development of technology from flow cytometry to single-cell transcriptomics, we have a more profound knowledge about uNK cell subtypes. Nowadays, uNK still have not been definitively linked to pregnancy pathologies, which may be associated with the subset complexity. Different uNK subsets have diverse cytokine production and KIRs, predicting different functions. More specific definition and description of uNK subsets may provide deeper insight into the functions of precise treatment for pregnancy complications.

3. The particular KIR/HLA combinations have either a protective or detrimental role in placentation, which can predict NK cell responses and possible treatment modifications. Understanding how the interaction between KIR and HLA genes has an effect on the pathogenesis of disease is a challenging problem. The KIR expression on uNK cells is initially stable. Upon encountering allogenic fetus or invading pathogens, KIR expression will be altered to maximize the balance between self-tolerance and protection against viral infection. Besides, the expression of HLA-G, C, E and F are different during three trimesters in the pregnancy and play an important role in regulating immune response. The study of uNK KIR receptors and their interaction with trophoblast HLA-G, C, E and F help clinicians to prevent pregnancy complications such as RPL and PE.

4. The mechanism of trophoblast invasion and the migration and apoptosis of VSMCs are of great value for our comprehensive understanding of SA remodeling, facilitating the early diagnosis and treatment of pregnant complications due to dysfunctional SA remodeling such as PE and PRL. Ma et al. firstly bring up a concept of migracytosis, a cell migration-dependent process for the release of intracellular contents through migrasome, which is a newly discovered organelle (155). Researchers have demonstrated that migrasomes play a critical role in cellular communication, cell migration and early embryo development. It is very likely that migrasomes function in immune response and angiogenesis (156–158). Whether migrasomes are produced by trophoblasts and VSMCs and whether migrasomes regulate trophoblast invasion and VSMC migration at the maternal-fetal interface during pregnancy are total brand new areas remaining to be explored. Migrasomes may also play a role in cell-cell communication between uNK cells and EVT, providing a new target for regulating SA remodeling.

Recently, spatial transcriptomics have developed greatly, which can provide specific locality information combined with high-resolution transcriptome profiles to deconvolute the cellular compositions of the maternal-fetal interface microenvironment. There is no way to elucidate the cellular interactions purely based on single-cell RNA-seq data due to the absence of spatial information. Li et al. used the mouse model to identify the cellular composition and the embryonic communication with maternal decidua during early pregnancy under the guidance of spatial transcriptome technology (159). Spatial transcriptome can be further employed in the human placenta during the first, second and third trimester, which facilitates the understanding of dynamic changes of KIRs and HLA and communication between uNK and EVT. A comprehensive understanding of the interaction between decidual NK cells and EVTs can provide us with new insights into the pathogenesis of RPL and PE, which urges us to explore more effective diagnostic methods and treatments for pregnancy complications resulting from SA remodeling.

## Author contributions

YL designed this study; X-WW and Y-CZ wrote the review; FW and F-JT revised the review. All authors contributed to the article and approved the submitted version.

## Funding

This work was supported by the National Key Research and Development Program of China (2018YFC1002803), the National Natural Science Foundation of China (82171669 to Yi Lin), and the Shanghai Jiao Tong University Trans-med Awards Research (Major Project) (20210201).

## Conflict of interest

The authors declare that the research was conducted in the absence of any commercial or financial relationships that could be construed as a potential conflict of interest.

## Publisher’s note

All claims expressed in this article are solely those of the authors and do not necessarily represent those of their affiliated organizations, or those of the publisher, the editors and the reviewers. Any product that may be evaluated in this article, or claim that may be made by its manufacturer, is not guaranteed or endorsed by the publisher.
